# Position statement of the Spanish Society of Pediatric Rheumatology on infection screening, prophylaxis, and vaccination of pediatric patients with rheumatic diseases and immunosuppressive therapies: Part 1 (screening)

**DOI:** 10.1007/s00431-022-04418-7

**Published:** 2022-03-08

**Authors:** Esmeralda Núñez Cuadros, Joan Calzada-Hernández, Daniel Clemente, Sara Guillén Martín, Laura Fernández Silveira, María José Lirola-Cruz, Alfredo Tagarro, Marisol Camacho Lovillo, Rosa María Alcobendas Rueda, Agustín López López, Miren Satrustegi Aritziturri, Cristina Calvo

**Affiliations:** 1 Pediatric Rheumatology Unit, UGC Pediatría, Hospital Regional Universitario de Málaga, Instituto de Investigación Biomédica de Málaga (IBIMA), Av. Arroyo de los Ángeles, s/n 29011, Málaga, Spain; 2 Unitat de Reumatologia Pediàtrica, Servei de Pediatria, Hospital Sant Joan de Déu, Esplugues de Llobregat, Barcelona, Spain; 3 Pediatric Rheumatology Unit, Hospital Infantil Universitario, Niño Jesús, Madrid, Spain; 4 Department of Paediatrics, Hospital Universitario de Getafe, CIBERINFEC ISCIII, Getafe, Madrid, Spain; 5 Servicio de Inmunología, Reumatología e Infectología Pediátricas, Hospital Universitario Virgen del Rocío, Sevilla, Spain; 6 Department of Paediatríc Rheumatology, Instituto Hispalense de Pediatría, Seville, Spain; 7 Pediatrics Department, Instituto de Investigación 12 de Octubre (imas12), Hospital Universitario Infanta Sofía, Universidad Europea, Madrid, Spain; 8 Department of Pediatric Rheumatology, La Paz Children’s Hospital, Madrid, Spain; 9 Department of Paediatrics, Hospital Universitario Puerta de Hierro, Majadahonda, Madrid, Spain; 10 Servicio de Pediatría, Hospital Universitario Donostia, San Sebastián, Spain; 11 Department of Pediatrics, Infectious and Tropical Diseases, Hospital Universitario La Paz, and La Paz Research Institute (IdiPaz). CIBERINFEC. ISCIII, Madrid, Spain; 12 Translational Research Network of Pediatric Infectious Diseases (RITIP), Madrid, Spain

**Keywords:** Infections, Screening, Prophylaxis, Vaccination, Immune-mediated rheumatic diseases, Consensus

## Abstract

This study provides practical recommendations on infection screening in pediatric patients with immune-mediated rheumatic diseases and immunosuppressive therapies. For this reason, a qualitative approach was applied. A narrative literature review was performed via Medline. Primary searches were conducted using Mesh and free texts to identify articles that analyzed data on infections and vaccinations in pediatric patients with immune-mediated rheumatic diseases and immunosuppressive therapies. The results were presented and discussed in a nominal group meeting, comprising a committee of 12 pediatric rheumatologists from the infections prevention and treatment working group of the Spanish Society of Pediatric Rheumatology. Several recommendations were generated. A consensus procedure was implemented via a Delphi process that was extended to members of the Spanish Society of Pediatric Rheumatology and Vaccine Advisory Committee of the Spanish Association of Pediatrics. Participants to the process produced a score ranging from 0 = totally disagree to 10 = totally agree. Agreement was considered if at least 70% of participants voted ≥ 7. The literature review included more than 400 articles. Overall, 63 recommendations were generated (21 on infection screening) voted by 59 pediatric rheumatologists and other pediatric specialists, all of them achieving the pre-established agreement level. The recommendations on screening cover all the procedures (serology, assessment of risk factors, and other clinical activities) connected with the screening for infections including tuberculosis; hepatitis A, B, and C viruses; measles; mumps; rubella; diphtheria; and other infections.

*Conclusion*: Screening for infections is an essential part of risk management in pediatric patients with immune-mediated rheumatic diseases and immunosuppressive therapies.

**Table:** 

**What is Known:**
• *Infectious diseases and related complications are a major cause of morbidity and mortality in patients with immune-mediated rheumatic diseases.*
• *At present, practical information on infectious prophylaxis in children with rheumatic diseases is limited, and often extrapolated from children with cancer.*
**What is New:**
• *In the absence of evidence, a literature review and a Delphi survey were conducted to establish a series of expert recommendations that would be useful in clinical practice, providing a practical and simple day-to-day approach to be used by pediatric rheumatologists.*

## Introduction

Infectious diseases and related complications are a major cause of morbidity and mortality in patients with immune-mediated rheumatic diseases. The increased risk of infection in this population is probably due to the immune effects of the disease itself, use of immunosuppressive drugs, comorbidities, medical/surgical procedures, as well as frequent clinic visits [1].

The prevention of the spread of infectious diseases is based on the adoption of different measures including the reduction of disease transmission and incidence and, hence, the relevance of prophylaxis and immunizations. This requires an exhaustive knowledge of infectious disease characteristics, including source and reservoir of infections, transmission dynamics, and susceptible hosts.

Like adults, pediatric patients with immune-mediated rheumatic diseases are at an increased risk of infections [2]. With current aggressive treatment strategies incorporating the early use of immunosuppressive therapies like biological drugs, susceptibility to infections, including opportunistic ones, increases further [3]. Besides, it is important to bear in mind that during the first years of life children are being vaccinated, and that the immunogenicity of vaccinations may be reduced because of the immunosuppressed status of these patients, thereby further increasing the infection risk [2]. Therefore, the screening for infection in patients with immune-mediated rheumatic diseases, who are scheduled to begin immunosuppressive treatment, is vital.

Considering the previously exposed, we designed this project to generate practical recommendations on infection screening, prophylaxis, and vaccination in pediatric patients with immune-mediated rheumatic diseases prior to immunosuppressive therapy initiation. This article describes the evidence found and relevant recommendations generated on infection screening in this population. We are confident this guide will help physicians resolve questions that may arise in day-to-day practice, thereby improving pediatric patient care and outcomes.

## Methods

This qualitative work was based on a comprehensive narrative literature review, the experience of an expert committee, and the consensus achieved among pediatric rheumatologists. The project was carried out following the Declaration of Helsinki’s ethical principles for medical research involving human subjects, and in accordance with the Good Clinical Practice regulations. The whole process was supervised by an expert methodologist. The project was promoted by the Spanish Society of Pediatric Rheumatology (SERPE).

First, a group of 12 pediatric rheumatologists from the infections prevention and treatment working group of SERPE was selected, 6 of them are also members of the Spanish Society of Pediatric Infections (SEIP), and all of them have recognized experience in the care of pediatric patients with immune-mediated rheumatic diseases.

### Literature review

With the help of an expert documentalist, a narrative literature review in Medline was performed using PubMed’s Clinical Queries tool, along with individual searches using Mesh and free text terms up to December 2020, which was then updated for publishing purposes in April 2021. We sought to identify articles describing screening, prophylaxis, and vaccinations in pediatric patients with rheumatic diseases using corticosteroids (CS) and classical synthetic and biologic disease-modifying antirheumatic drugs (cs and bDMARDs). More specifically, the inclusion criteria were (1) pediatric patients (≤ 18 years); (2) articles analyzing any aspects related to screening, prophylaxis, and vaccinations in patients scheduled to begin therapy with CS, csDMARDs, and bDMARDs; (3) no restrictions concerning the comparator (whether it existed or not); (4) meta-analyses, systematic literature reviews (SLRs), randomized clinical trials, and observational studies. Two reviewers independently selected the articles, first by title and abstract, then by reading the full article in detail; they both collected data. In order to avoid duplicates, randomized clinical trials or observational studies were only included if they were not in the meta-analyses or SLRs. Evidence and result tables were generated. Study quality was assessed using the 2011 Oxford Scale [4].

### Nominal group meeting

A nominal group meeting was held by the expert committee during which they first defined the objectives, scope, and users of the document. Through a guided discussion, the experts then discussed the available evidence based on the review; they addressed all aspects related to the screening prior to CS and bDMARD initiation, and to infectious prophylaxis. This resulted in the generation of several recommendations.

### Delphi process

With all the previously described information, a series of preliminary recommendations were proposed. After several revisions by the experts, the definitive recommendations were generated, which were subsequently submitted to an online Delphi vote. In addition, the Delphi process was extended to a group of 92 experts in their field, from the SERPE and SEIP. Participants voted each recommendation, using a scale ranging from 1 to 10 (1 = strongly disagree to 10 = strongly agree). Agreement was defined if at least 70% of participants voted ≥ 7. Recommendations with a level of agreement (GA) below 80% were re-evaluated and, if appropriate, reworded; they then underwent a second round of voting.

### Final consensus document

After the Delphi process and along with the results of the literature review, the final document was drafted. This current part corresponds to infection screening. With the assistance of a methodologist, each recommendation was assigned a level of evidence (LE) and grade of recommendation (GR) according to the recommendations for evidence-based medicine of the Oxford Center for Evidence-Based Medicine [4]. The GA were assigned, as described above. The final document was reviewed by the expert committee of the working group on prevention of infections in children with rheumatic diseases of the Spanish Society of Pediatric Rheumatology, who drafted the final comments.

## Results

The recommendations generated in this consensus document, as well as the Delphi process results, are depicted in Table 1. A total of 59 experts participated in the Delphi (response rate 64%), 45 from SERPE and 14 form SEIP.

**Table 1:** Delphi results

#	Recommendation	Mean	SD	Median	p25	p75	Min	Max	LE	GR	GA
1	Screening for tuberculosis infection should be performed before initiating immunosuppressive therapy	9.46	4.24	10	10	10	4	10	IIIb	B	96%
2	Screening for TB infection should include a directed medical history, physical examination, and tuberculin skin test	9.40	1.28	10	9.25	10	4	10	IIa	B	96%
3	Along with the TST, interferon-γ release assays should be performed in the following groups: • Children aged ≤ 5 years; • BCG vaccination; • Patients on CS; • Patients in whom the underlying disease encompasses a baseline inflammatory state (especially with elevated acute phase reactants); • Patients initiating biological therapy In the rest of patients, interferon-γ release assays are recommended, but not mandatory	9.04	1.42	10.00	8.00	10	5	10	IIIa	B/C	94%
4	Either of the IGRAs, QuantiFERON® TB-Gold In Tube assay (Cellestis/Qiagen, Carnegie, Australia) or T-SPOT®.TB assay (Oxford—Immunotec, Abingdon, UK), can be used	8.40	1.86	9.00	8.00	10	5	10	IIIa	B	81%
5	In cases of IGRA indeterminate results: (1) TST should be performed (if previously not performed); (2) IGRA test should be repeated (using the same or a different assay); (3) assessment of the likelihood of TB should be individualized according to patient’s risk factors	9.26	0.95	10	9	10	7	10	IIIa	C	100%
6	A chest X-ray should be performed in (1) patients with TB symptoms; (2) asymptomatic patients with a positive screening test for TB infection; (3) asymptomatic patients with a negative screening test for TB infection, yet with recent and close contact with a known active TB patient or other risk factors for TB infection; (4) patients with an indeterminate IGRA test result	9.31	0.71	10	9	10	6	10	IIIa	B	98%
7	Active TB contacts, TB risk factors, and TB clinical symptoms and signs should be assessed regularly through anamnesis and physical examination	8.98	1.31	10	8	10	5	10	IIIb	C	96%
8	When a new risk factor for TB infection during immunosuppressive treatment is detected, a TST and at least one IGRA assay should be performed	9.41	1.08	10	9	10	6	10	IIa	B	98%
9	Serological screening for *Trypanosoma cruzi* infection should be performed in children with rheumatological diseases originating themselves from endemic areas, or whose mothers originate from endemic areas without any specific serology performed during pregnancy	8.64	1.70	9	8	10	5	10	IIIb	C	82%
10	Measles, mumps, and rubella serologic screening should be performed before initiating immunosuppressive treatment in patients with incomplete vaccination status or doubts about vaccination. However, regular serological screening is not recommended	8.56	1.97	9	8	10	1	10	IIa	B	88%
11	Currently, tetanus and diphtheria serologic screening is not recommended in patients starting immunosuppressive therapy	9.28	1.53	10	9	10	1	10	IIa	B	94%
12	Anti-HBs serology should be performed in all patients before initiating immunosuppressive therapy. If anti-HBs titers < 10 mIU/mL, revaccination is indicated	9.08	6.36	10	9	10	1	10	IIa	B	90%
13	It is not recommended to repeat HBV serology in children if HBV titers are ≥ 10 mIU/mL. In revaccination cases, seroconversion should be checked	8.58	3.54	10	7	10	4	10	IIa	B	86%
14	Serologic screening for HBV should be performed in unvaccinated children or those with risk factors, regardless of the treatment scheduled	8.48	2.29	10	8	10	2	10	IIa	B	82%
15	Hepatitis A virus vaccine is recommended in children. There is no evidence for recommending a prior serologic screening	8.42	3.54	9	7	10	2	10	IIIa	B	86%
16	Routine serologic screening for hepatitis C virus in pediatric populations is not recommended. It should, however, be performed in patients with risk factors or in the event of transaminase level elevations	7.68	2.85	9	5.25	10	1	10	IIIb	C	72%
17	Varicella serologic screening should be performed in all pediatric patients with rheumatic diseases without any previous history of varicella or herpes zoster, nor vaccination or immunity evidence in a previous serology testing	9.24	1.36	10	9	10	4	10	IIIb	C	96%
18	Performing serologic screening for *Strongyloides stercoralis* infection is desirable in children from endemic areas, especially if they present with eosinophilia	8.51	4.77	9	7	10	4	10	IIIb	D	82%
19	Before initiating RTX treatment, it is recommended to assess immunoglobulin levels and lymphocyte subpopulations. If low immunoglobulin levels are recorded (according to patient’s age), assessments should be repeated periodically during RTX treatment	9.27	1.41	10	9	10	5	10	IIIb	D	96%
20	It is recommended to monitor the number and severity of infections during RTX treatment in order to identify patients possibly requiring immunoglobulin replacement therapy	9.51	0.97	10	9	10	5	10	IIIb	D	98%
21	Pre-treatment blood count assessment is recommended to detect neutropenia and eventually pursue hematological tests. However, the optimal frequency of hematological testing has not yet been established during or after RTX treatment	9.16	1.17	10	9	10	4	10	IIIa	C	94%

The level of evidence was assessed with the Oxford Center for Evidence-Based Medicine classification

*SD* standard deviation, *Min* minimum, *Max* maximum, *LE* level of evidence, *GR* grade of recommendation, *GA* grade of agreement, *IGRA* interferon-γ release assay, *TST* tuberculin skin test, *BCG* Bacille Calmette-Guérin, *CS* corticosteroids, *TB* tuberculosis, *anti-HBs* antibodies to hepatitis B surface antigen, *mIU/mL* milli-international units per milliliter, *HBV* hepatitis B virus, *RTX* rituximab

*Recommendation 1. Screening for tuberculosis infection should be performed before initiating immunosuppressive therapy (LE IIIa; GR B; GA 96%)*

The Center for Disease Control and Prevention (CDC) recommends screening for latent tuberculosis infection (TB) in all patients starting immunosuppressive therapy, including tumor necrosis factor (TNF) inhibitors and oral chemotherapy drugs like methotrexate [5].

The TB risk is higher for patients receiving TNF inhibitors, being the highest for infliximab followed by adalimumab and etanercept. The position of certolizumab pegol and golimumab on this risk scale is still unclear [6, 7].

Although the risk of TB is uncertain, screening is also recommended for patients who are to receive anti-IL1 (anakinra/canakinumab), anti-IL-6 (tocilizumab), or rituximab.

*Recommendation 2. Screening for TB infection should include a directed medical history, physical examination, and tuberculin skin test or interferon-γ release assay (IGRA) (NE IIa; GR B; GA 96%)*

Screening for TB infection should include specific questions, with special attention directed to TB cardinal symptoms and TB risk factors (place of birth or residence, previous TB, known active TB contacts, visits to TB endemic countries, or immunosuppressive treatment) [8–15].

A test to demonstrate contact with *Mycobacterium tuberculosis* should also be performed, using either the tuberculin skin test (TST) or IGRA {Vassilopoulos, 2011 #15;Behar, 2009 #16;Detjen, 2007 #17;Winthrop, 2018 #152}.

The screening should also be adapted to the epidemiological situation, population characteristics, disease and treatments of the patient, and according to the accessibility of IGRA tests and tests’ probability of false-negatives or false-positives.

*Recommendation 3. Along with the TST, interferon-γ release assays should be performed in the following groups:*

- *Children aged* ≤ *5 years;*
- *Bacille Calmette-Guerin (BCG) vaccination;*
- *Patients on CS;*
- *Patients in whom the underlying disease encompasses a baseline inflammatory state (especially with elevated acute phase reactants);*
- *Patients initiating biological therapy.*

*In the rest of patients, interferon-γ release assays are recommended, but not mandatory (NE IIIa; GR B/C; GA 94%)*

Several studies have revealed a higher incidence of indeterminate IGRA test results in patients aged 5 years or below. As a consequence, CDC recommendations prefer TST to be carried out in these patients. Taking into consideration the epidemiological situation of Spain, dual screening using TST and IGRAs is thus recommended, in order to increase the screening sensitivity [19–21].

BCG vaccination can produce false-positives in the TST. On the other hand, antigens that elicit immune responses in IGRAs are absent in BCG; consequently, IGRAs display superior specificity for *Mycobacterium tuberculosis* infection compared with the TST in individuals with a history of BCG vaccination [18, 20, 22, 23].

CS are associated with false-negatives in the TST [20, 24], and the rate of indeterminate results with IGRAs increases in patients with baseline inflammatory status [25–27]. Therefore, in both cases, a dual screening is recommended.

Finally, there is substantial uncertainty among clinicians concerning the management of patients undergoing biological therapies and the best strategies to adopt for TB prevention. As in the previous cases, both tests are thus recommended.

*Recommendation 4. Either of the IGRAs, QuantiFERON* ^*®*^ *TB-Gold In Tube assay (Cellestis/Qiagen, Carnegie, Australia) or T-SPOT®.TB assay (Oxford- Immunotec, Abingdon, UK), can be used (LE IIIa; GR B; GA 81%)*

Only a few studies have directly compared QuantiFERON^®^ and T-SPOT^®^.TB, showing no clear differences [28–31].

*Recommendation 5. In cases of IGRA indeterminate results: (1) TST should be performed (if previously not performed); (2) IGRA test should be repeated (using the same or a different assay); (3) assessment of the likelihood of TB should be individualized according to patient’s risk factors (LE IIIa; GR C; GA 100%)*

As the indeterminate result in the IGRA might be due to inflammatory activity, the repetition of the same assay should be considered if this situation changes in any way. Otherwise, a change of the IGRA assay could be considered [14].

*Recommendation 6. A chest X-ray should be performed in (1) patients with TB symptoms; (2) asymptomatic patients with a positive screening test for TB infection; (3) asymptomatic patients with a negative screening test for TB infection, yet with recent and close contact with a known active TB patient or other risk factors for TB infection; and (4) patients with an indeterminate IGRA test result (LE IIIa; GR B; GA 98%)*

In cases of indeterminate IGRA and negative TST, risk assessment should be conducted on an individual basis. A chest radiograph is highly recommended, especially in patients with risk factors for false-negative TST results.

A recent and close contact is defined as sharing > 4 h per day in the same enclosed room with a confirmed/suspected TB patient (pulmonary, laryngeal, tracheal, or endobronchial TB) within the last 3 months [32, 33].

*Recommendation 7. Active TB contacts, TB risk factors, and TB clinical symptoms and signs should be assessed regularly through anamnesis and physical examination (LE IIIb; GR C; GA 96%)*

TB risk factors currently considered include close and recent contact with a suspected or confirmed case of active TB, recent travel to a region with high TB prevalence, injecting drug use, children aged ≤ 5 years, diseases or conditions associated with immune system alterations, such as chronic renal failure, cancer, primary, or secondary immunodeficiencies, and use of immunosuppressive drugs [20, 34].

*Recommendation 8. When a new risk factor for TB infection during immunosuppressive treatment is detected, a TST and at least one IGRA assay should be performed (LE IIa; GR B; GA 98%)*

Different position statements are in line with this recommendation [9, 10, 12–18]. Sensitivity of TST is reduced in children receiving immunosuppressive treatment, with a high percentage of false negatives; hence, at least one IGRA technique (ideally, in parallel with TST) should be performed to maximize diagnostic performance.

*Recommendation 9. Serological screening for Trypanosoma cruzi infection should be performed in children with rheumatological diseases originating themselves from endemic areas, or whose mothers originate from endemic areas without any specific serology performed during pregnancy (LE IIIb; GR C; GA 82%)*

According to the World Health Organization, Chagas disease is endemic in 21 Latin American countries [35], and most infected patients display no symptoms in either the acute or chronic disease phase [36].

In immunocompromised patients, Chagas disease reactivation may occur and exhibit severe manifestations like meningitis, encephalitis, or myocarditis. In autoimmune diseases, Chagas disease reactivation is rare, but it has also been associated with immunosuppressive therapy [37].

The treatment of the disease in its early stages (acute phase) is more effective than in the chronic phase. Therefore, it is essential to diagnose Chagas disease early on [36]. In patients with chronic pauci-symptomatic disease, Chagas disease reactivation may occur. Should this be the case, myocarditis and meningoencephalitis are particularly severe, and common, as well [38].

*Recommendation 10. Measles, mumps, and rubella serologic screening should be performed before initiating immunosuppressive treatment in patients with incomplete vaccination status or doubts about vaccination. However, regular serological screening is not recommended (LE IIa; GR B; GA 88%)*

A study involving 4–8-year-olds having undergone measles, mumps, and rubella (MMR) vaccination during the second year of life reported a high sero-protection rate for measles after two doses (90.2%), with a slightly lower rate (87.7%) after a single dose. Antibody concentrations were strongly dependent on the period since the last vaccination [39]. Concerning mumps, sero-protection rates were 74.4% after two doses, and 59% after a single one. The antibody titer was more influenced by the number of doses than the time interval since the last vaccination [39]. As regards rubella, following two doses of MMR vaccine, 68% of girls and 58% of boys were reported to maintain antibody titers of 1:32 or higher. The determining factor of antibody level was the time interval since the last vaccination [39].

Another study involving 43 children having undergone MMR vaccinations at 9 and 15 months of age revealed sero-protection rates of 80%, 85%, and 96% for measles, mumps, and rubella, respectively, when the children were aged 4–6 years, and of 83%, 96.7%, and 96.7% when they were between 9 and 12 years of age. Similar results were reported in other articles [40–42].

An average decrease in antibody levels of 7.4% per year for measles, 5.7% for rubella, and 9.2% for mumps was reported. The rate of decline is higher in patients with lower post-vaccination antibody levels, and in those with increased antibody levels less than two times, as compared to pre-vaccine antibody values [43]. In this context, other reports observed persistent seroconversion rates for measles, mumps, and rubella in 95%, 74%, and 100% of MMR-vaccinated patients, 15 years after receiving the second MMR dose [44].

According to one article, however, juvenile idiopathic arthritis (JIA) patients may present with lower sero-protection rates and antibody levels for measles, rubella, mumps, and diphtheria than healthy controls [45]. Yet, these data should be interpreted with care, given that the control group was an historical cohort. This paper’s authors recommend performing serological tests periodically, in order to detect any immune status changes of JIA patients [45].

*Recommendation 11. Currently, tetanus and diphtheria serologic screening should be performed before initiating immunosuppressive treatment in patients with incomplete vaccination status or doubts about vaccination. However, regular serological screening is not recommended (LE IIa; GR B; GA 94%)*

In an Austrian study [39] involving 338 subjects aged 4–8 years, more than 80% of children achieved a protective level following the recommended immunization schedule for diphtheria and tetanus. Antibody concentration strongly depended on the period since the last vaccination.

Another study that assessed the serostatus of healthy children aged 11–13 years prior to second booster administration (the first was administered at 6 years) reported that 98% of children exhibited antibody levels for diphtheria > 0.01 IU/mL, with 63% of them displaying antibody levels > 0.1 IU/mL, as well as 100% of children exhibiting antibody levels for tetanus > 0.01 IU/mL, with 96% of them displaying antibody levels > 0.1 IU/mL [46].

*Recommendation 12. Anti-HBs serology should be performed in all patients before initiating immunosuppressive therapy. If anti-HBs titers < 10 mIU/mL, revaccination is indicated (LE IIa; GR B; GA 90%)*

Long-term protection against hepatitis B virus (HBV) with current vaccines remains unknown [39, 47–50]. One study showed that 15 months after third dose administration of two hexavalent vaccines, antibodies to hepatitis B surface antigen (anti-HBs) titers ≥ 10 mIU/mL varied from 69% (Hexavac) to 96% (Infanrix) [47]. After a booster dose, 93% of children achieved titers ≥ 10 mIU/mL, without differences between groups. Another report analyzed 326 children aged 4–8 years, most of whom had received Hexavac vaccination. In this study, antibody titers above 10 mIU/mL were present in only 52% of children [39].

*Recommendation 13. It is not recommended to repeat HBV serology in children if HBV titers are ≥ 10 mIU/mL. In revaccination cases, seroconversion should be checked (LE IIa; GR B; GA 86%)*

Based on vaccine efficacy studies, sero-protection against HBV infection is defined as anti-HBs level ≥ 10 mIU/mL after receiving a complete immunization schedule [51]. Based on current scientific evidence, booster vaccination against HBV for immunocompetent children and adults is not recommended for long-term protection [51, 52]. Immunocompromised patients, however, should be monitored and receive a booster vaccination if their anti-HBs levels decrease < 10 mIU/mL [51].

As exposed before, there could be differences in HBV immunity depending on the vaccine. In general, Infanrix vaccinations have depicted better seroconversion rates than Hexavac [47]. In September 2005, the European Medicine Agency (EMA) recommended the discontinuation of Hexavac vaccine due to concerns regarding long-term immunogenicity. Infanrix hexa was recommended instead [47, 53].

*Recommendation 14. Serologic screening for HBV should be performed in unvaccinated children or those with risk factors, regardless of the treatment scheduled (LE IIa; GR B; GA 82%)*

HBV risk factors include adolescents with sexual activity or high-risk behaviors, such as injecting drug use, history of sexually transmitted infections, children born to hepatitis B surface antigen (HBs-Ag)–positive mothers, diseases or conditions associated with immune system alteration, such as chronic renal failure, cancer, and primary or secondary immunodeficiencies, and use of immunosuppressive drugs. Vertical transmission is the primary route of transmission of viral hepatitis in children [54].

The occurrence of HBV infection in vaccinated populations varies across countries and studies. A report from the Czech Republic involving newborns (to HBsAg-positive mothers) that received hepatitis B immunoglobulins and a complete vaccination schedule depicted an anti-HBc seroconversion rate of 1.5% in children aged 3–5 years, and of 8% in children over 15 years of age [55]. Data from other countries revealed an anti-HBc seroconversion rate of 1.7% in the UK, 7.5% in Iran, 8.9–33.3% in China, or 4.1% in Taiwan [51].

On the other hand, in a cohort of immunized children starting at birth with a three-dose regimen of HBV vaccine, these children received a booster dose 10 and 15 years after vaccination. Among 108 participants who had lost protective antibody levels against HBV, more than 70% exhibited an anamnestic response (defined as a rapid prominent increase in antibody levels following second contact with the antigen) to the booster dose [56]. Therefore, some authors recommend a HBV vaccine booster administration, as based on the patients’ serological status [48, 51].

The Vaccine Advisory Committee of the Spanish Association of Pediatrics and Spanish Society of Pediatric Rheumatology recommend antibody assessment in all immunocompromised patients, along with the administration of a booster dose if anti-HBs titers are ≤ 10 mIU/mL [57]. Some guidelines consider anti-HBs titers ≥ 100 mIU/mL an ideal sero-protection value [58].

*Recommendation 15. Hepatitis A virus vaccine is recommended in children. There is no evidence for recommending a prior serologic screening (LE IIIa; GR B; GA 86%)*

The hepatitis A virus (HAV) incidence rate in Spain was estimated at 1.32 per 100,000 inhabitants in 2013, with a seroprevalence of 20% in children aged 10 years in 2000, according to the WHO [57]. A study conducted in Madrid region (Spain) in 2008–2009 revealed a 13.5% seroprevalence in children aged 13 years [59].

HAV has been suggested to possibly induce serious complications, such as the macrophage activation syndrome in patients with JIA [60].

Taking into account the high HAV prevalence and its potential complications, main national clinical guidelines recommend HAV vaccination, along with periodic testing of antibody titers [57].

*Recommendation 16. Routine serologic screening for hepatitis C virus in pediatric populations is not recommended. It should, however, be performed in patients with risk factors or in the event of transaminase level elevations (LE IIIb; GR C; GA 72%)*

The prevalence of hepatitis C virus (HCV) infection is very low in children with rheumatic diseases [61]. Thus, mass screening is not recommended. However, physicians should be aware of HCV risk factors, particularly adolescents with sexual activity or high-risk behaviors, such as injecting drug use, history of sexually transmitted infections, and children born to HCV-positive mothers. In these cases, and particularly in the presence of abnormal hepatic laboratory values, a screening should be considered.

*Recommendation 17. Varicella serologic screening should be performed in all pediatric patients with rheumatic diseases without any previous history of varicella or herpes zoster, nor vaccination or immunity evidence in a previous serology testing (LE IIIb; GR C; GA 96%)*

Biologic therapies in adults with rheumatoid arthritis are associated with a small but significant risk of specific opportunistic infections [62]. In the Spanish BIOBADASER registry, varicella zoster infections were the most common viral infections [63].

The safety of biologics in pediatric patients is acceptable. The rate of serious infections is very low, yet biological agents are associated with a higher risk of infection compared with methotrexate. This infection risk appears to be lower with abatacept, adalimumab, and etanercept [64].

Therefore, screening for varicella infection is recommended before initiating immunosuppressive treatment in children.

*Recommendation 18. Performing serologic screening for Strongyloides stercoralis infection is desirable in children from endemic areas, especially if they present with eosinophilia (LE IIIb; GR D; GA 82%)*

Residence or recent travel to tropical areas, especially Southeast Asia, sub-Saharan Africa, and Latin America, has been described as a risk factor for *Strongyloides stercoralis* infection. In Spain, isolated cases have been reported, with the exception of the Valencian coast that is considered endemic [65].

*Recommendation 19. Before initiating RTX treatment, it is recommended to assess immunoglobulin levels and lymphocyte subpopulations. If low immunoglobulin levels are recorded (according to patient’s age), assessments should be repeated periodically during RTX treatment (LE IIIb; GR D; GA 96%)*

Prolonged and total B-cell depletion induced by anti-CD20 agents like RTX may cause hypogammaglobulinemia and neutropenia [66–68]. Based on available data, anti-CD20 agents are associated with at least a modest increase risk for infections. However, not all patients that develop hypogammaglobulinemia are at increased risk of developing infection after B-cell depleting therapy [69].

Concerning RTX retreatment in patients having developed neutropenia, those with mild self-limited neutropenia can successfully undergo repeated RTX treatment. However, there is evidence that neutrophil counts < 500/mm^3^ may be associated with severe infection, thus requiring treatment using intravenous antibiotics and granulocyte colony-stimulating factor [66–69].

Anti-*Pneumocystis jirovecii* prophylaxis in patients on RTX should be considered only in those on concomitant therapies associated with an increased risk for *Pneumocystis jirovecii* infection, such as CS (prednisone 20 mg daily or equivalent doses for at least 4 weeks) [66–69].

Serologic screening for chronic or resolved HBV infection should be performed before initiating anti-CD20 agents; in order to prevent HBV reactivation, antiviral prophylaxis should be continued during anti-CD20 treatment and for at least 12–18 months after the last dose in HBsAg-positive patients. Monitoring of HBV reactivation should be carried out for at least 12 months after completing antiviral prophylaxis. Prophylaxis should be offered to HBsAg-negative/anti-HBc-positive patients so as to prevent reactivation of a resolved HBV infection [69].

*Recommendation 20. It is recommended to monitor the number and severity of infections during RTX treatment, in order to identify patients possibly requiring immunoglobulin replacement therapy (LE IIIb; GR D; GA 98%)*

A minority of patients on RTX are likely to develop severe hypogammaglobulinemia complicated by recurrent infection. Considering the pre-existing hypogammaglobulinemia-induced increased infection risk on RTX treatment, immunoglobulin levels should be assessed prior to RTX initiation and, if immunoglobulin levels are low, they should be monitored during treatment [68, 70]. Monitoring is also indicated in patients with severe infectious diseases so as to identify those possibly requiring immunoglobulin replacement therapy.

When considering immunoglobulin replacement therapy, the following factors should be considered: severity of IgG deficiency, presence of recurrent infections (especially sinopulmonary), and lack of normal responses to tetanus and pneumococcal vaccines [66, 68, 70].

Recommendation 21. Pre-RTX treatment blood count assessment is recommended to detect neutropenia and eventually pursue hematological tests. However, the optimal frequency of hematological testing has not yet been established during or after RTX treatment (LE IIIa; GR C; GA 98%)

As RTX is associated with neutropenia [71], it is recommended to perform a hematological test before RTX treatment initiation, followed by regular controls.

## Discussion

In this paper, we present a series of recommendations concerning the screening for different infections in children with rheumatic diseases prior to initiating immunosuppressive drugs (including biologics), based on the best available evidence.

For this purpose, the nominal group and Delphi methodology that is widely used in this type of documents has been followed. In addition, along with a review of the available evidence, a group of experts in the field was selected for the drafting of the recommendations.

In this document, we strongly emphasize the relevance of a good epidemiological clinical history, along with an exhaustive physical examination, which helps recognizing symptoms and suspecting different diseases, in addition to performing the screening tests.

The recommendations are intended to assist specialists involved in the care of these patients in their routine clinical practice. In addition, there is no doubt that the availability of explicit recommendations covering all aspects of infection screening in relation with immunosuppressive treatments is an essential element of good clinical practice, as demonstrated in this document.

Data from pharmacovigilance cohorts will be useful for actual risk monitoring in clinical practice [11, 72, 73].

## Data Availability

N/A.

## Code availability

N/A.
