# Correction to: Position statement of the Spanish society of pediatric rheumatology on infection screening, prophylaxis, and vaccination of pediatric patients with rheumatic diseases and immunosuppressive therapies: part 1 (screening)

**DOI:** 10.1007/s00431-022-04448-1

**Published:** 2022-03-21

**Authors:** Esmeralda Núñez Cuadros, Joan Calzada-Hernández, Daniel Clemente, Sara Guillén Martín, Laura Fernández Silveira, María José Lirola-Cruz, Alfredo Tagarro, Marisol Camacho Lovillo, Rosa María Alcobendas Rueda, Agustín López López, Miren Satrustegi Aritziturri, Cristina Calvo

**Affiliations:** 1 Pediatric Rheumatology Unit, UGC Pediatría, Hospital Regional Universitario de Málaga, Instituto de Investigación Biomédica de Málaga (IBIMA), Av. Arroyo de los Ángeles, s/n 29011, Málaga, Spain; 2 Unitat de Reumatologia Pediàtrica, Servei de Pediatria, Hospital Sant Joan de Déu, Esplugues de Llobregat, Barcelona, Spain; 3 Pediatric Rheumatology Unit, Hospital Infantil Universitario Niño Jesús, Madrid, Spain; 4 Department of Paediatrics, Hospital Universitario de Getafe, CIBERINFEC. ISCIII, Getafe, Madrid, Spain; 5 Servicio de Inmunología, Reumatología E Infectología Pediátricas, Hospital Universitario Virgen del Rocío, Sevilla, Spain; 6 Department of Paediatríc Rheumatology, Instituto Hispalense de Pediatría, Seville, Spain; 7 Pediatrics Department, Instituto de Investigación 12 de Octubre (imas12), Hospital Universitario Infanta Sofía, Universidad Europea, Madrid, Spain; 8 Department of Pediatric Rheumatology, La Paz Children’s Hospital, Madrid, Spain; 9 Department of Paediatrics, Hospital Universitario Puerta de Hierro, Majadahonda, Madrid, Spain; 10 Servicio de Pediatría, Hospital Universitario Donostia, San Sebastián, Spain; 11 Department of Pediatrics, Infectious and Tropical Diseases, Hospital Universitario La Paz, and La Paz Research Institute (IdiPaz). CIBERINFEC. ISCIII, Madrid, Spain; 12 Translational Research Network of Pediatric Infectious Diseases (RITIP), Madrid, Spain

## Correction to: European Journal of Pediatrics https://doi.org/10.1007/s00431-022-04418-7

In the original published version of this article, the affiliation of the co-authors Sara Guillén Martín^4^, Laura Fernández Silveira^5^, Alfredo Tagarro^7^ and Marisol Camacho Lovillo^5^ has been updated as follows:

^4^Department of Paediatrics, Hospital Universitario de Getafe, CIBERINFEC. ISCIII, Getafe, Madrid, Spain.

^5^Servicio de Inmunología, Reumatología e Infectología Pediátricas, Hospital Universitario Virgen del Rocío, Sevilla, Spain.

^7^Pediatrics Department, Instituto de Investigación 12 de Octubre (imas12), Hospital Universitario Infanta Sofía, Universidad Europea, Madrid, Spain.

The original article has been corrected.

